# Tongue somatosensory evoked potentials reflect midbrain involvement in patients with clinically isolated syndrome

**DOI:** 10.3325/cmj.2016.57.558

**Published:** 2016-12

**Authors:** Magdalena Krbot Skorić, Ivan Adamec, Luka Crnošija, Tereza Gabelić, Barbara Barun, Ivana Zadro, Silva Butković Soldo, Mario Habek

**Affiliations:** 1 University Hospital Center Zagreb, Department of Neurology, Referral Center for Autonomic Nervous System Disorders, Zagreb, Croatia; 2 School of Medicine, University of Zagreb, Zagreb, Croatia; 3 University Hospital Centre Osijek, Department of Neurology, Osijek, Croatia

## Abstract

**Aim:**

To test the hypothesis that tSSEP findings reflect clinical and MRI MS lesions, the aim of this study was to investigate tSSEP changes in patients with clinically isolated syndrome (CIS) in relation to clinical and brainstem MRI findings. The second aim was to investigate whether the interpretation of the tSSEP results in the form of the tSSEP score enables better evaluation of the afferent trigeminal pathway involvement than analyzing each tSSEP parameter separately.

**Methods:**

115 consecutive CIS patients were enrolled from August 1, 2014 until March 1, 2016. Facial sensory symptoms and brainstem MRI (1.5 T) lesions were analyzed. tSSEP testing was performed for each patient from the raw tSSEP data. The tSSEP score was calculated separately for the left and right side (according to the cut-off values for absent response and prolonged latency of the main component, P1 (0 = normal response, 1 = prolonged latency, 3 = absent response) and the two values were summed.

**Results:**

There was no difference in the absolute values of the tSSEP variables regarding the presence of clinical symptoms. No association was found between tSSEP abnormalities and clinical symptoms (*P* = 0.544). Brainstem lesions (midbrain and pons) were associated with the absent tSSEP responses (*P* = 0.002 and *P* = 0.005, respectively). tSSEP score was significantly higher in patients with brainstem lesions (*P* = 0.01), especially midbrain (*P* = 0.004) and pontine (*P* = 0.008) lesions. Binary logistic regression showed that tSSEP score had a significant effect on the likelihood that patients have midbrain MR lesions, χ^2^(1) = 6.804, *P* = 0.009; and the model correctly classified 87% of cases.

**Conclusions:**

The consistent finding of this study was the association between tSSEP and midbrain lesions on MRI, indicating that tSSEP evaluates proprioception of the face. This study establishes the value of tSSEP in assessing brainstem function in early multiple sclerosis.

Clinically isolated syndrome (CIS) is an acute or subacute episode of neurologic deficit and is a presenting syndrome in 85% of patients who will ultimately develop multiple sclerosis (MS). Most of CIS patients present with optic neuritis, transverse myelitis, or brainstem/cerebellar symptoms, although a substantial number have multifocal symptoms (1). The affection of the brainstem in MS bears significant clinical importance. Patients with demyelinating lesions in the brainstem have a greater chance of having disability at follow-up than patients with no infratentorial lesions (2). Furthermore, they tend to have worse long term prognosis, and identification of these patients can influence important management decisions (3). Standard magnetic resonance imaging (MRI) of 1.5 Tesla field strength lacks sensitivity in detection of demyelinating lesions in the posterior fossa (4). Although MRIs of greater field strength, such as 3 or 7 Tesla, improve this shortcoming, the use of such machines is not yet widespread, and 7 Tesla MRI is mainly confined to research centers.

A common finding in MS patients are sensory symptoms (numbness, dysesthesia, or paresthesia) in trigeminal nerve regions (5). The trigeminal nerve is the most commonly involved isolated cranial nerve in MS (6). Interestingly, sensory symptoms associated with the trigeminal nerve damage in MS were found to be the only brainstem-specific symptoms negatively associated with the conversion to MS (7). Another problem with the trigeminal nerve involvement in MS is that even though high-resolution MRI at 3T yields a high prevalence of detectable trigeminal abnormalities, they did not correspond to trigeminal symptoms (8).

Several studies have been undertaken to improve the detection of nervous system impairment in MS using evoked potentials (9,10). Initial studies using brainstem trigeminal evoked potentials proved them to be more sensitive than MRI in revealing brainstem lesions (11). In our earlier work, we have investigated the potential of tongue somatosensory evoked potentials (tSSEP) as an important part of neurophysiological brainstem evaluation in MS patients (12,13).

In order to test the hypothesis that tSSEP findings reflect clinical and MRI MS lesions, the aim of this study was to investigate tSSEP changes in patients with clinically isolated syndrome (CIS) in relation to clinical and brainstem MRI findings. Furthermore, we wanted to investigate whether the interpretation of the tSSEP results in the form of the tSSEP score enables better evaluation of the afferent trigeminal pathway involvement than analyzing each tSSEP parameter separately.

## Materials and methods

This study included consecutive patients diagnosed with CIS from August 1, 2014 until March 1, 2016 at the Department of Neurology, University Hospital Center Zagreb. CIS diagnosis was made in patients with acute or subacute development of neurological symptoms and/or signs lasting longer than 48 hours in the absence of fever or infection, and with at least one demyelinating lesion larger than 3 mm on the brain and/or spinal cord MRI. The exception were patients with optic neuritis who were included if the classical clinical triad was present (rapidly developing impairment of vision, dyschromatopsia, and retro-orbital pain), accompanied by prolonged latencies of visual evoked potentials, irrespective of the presence of brain and/or spinal cord MRI lesion.

All patients were recruited after the MRI was performed and CIS diagnosis made. tSSEP testing was performed on the day of recruitment (median 35 days after the MRI, range from 0 to 206). The study was approved by the Ethics Committees of the University Hospital Center Zagreb and University of Zagreb, School of Medicine. All participants signed the informed consent.

### Clinical symptoms

All symptoms and signs pertaining to trigeminal involvement were recorded. These consisted of face and/or tongue pain, paresthesia, or hypoesthesia. The side of the face or tongue on which the symptoms were present was recorded as well.

### Tongue somatosensory evoked potentials (tSSEP)

tSSEP was performed according to previously published methods (12). Stimulation was delivered using modified EEG electrodes, located on the lateral side of the first two thirds of the tongue, and each side of the tongue was stimulated twice with 300 trials. Twister constant current stimulator was also used (Dr Langer Medical GmbH, Waldkirch, Germany). The frequency of the stimulation was 3 Hz and the duration of each stimulus was 0.2 ms. The cortical response was recorded from two surface disk electrodes situated on the contralateral side of the scalp, according to the International 10/20 system, at the middle position between C3 and T3 for the stimulation of the right side of the tongue – C5 electrode; and at the middle position between C4 and T4 for the stimulation of the left side of the tongue – C6 electrode and referred to the frontal electrode (Fz). The responses were recorded using a Brain Products Vision Recorder (Brain Products GmbH, Munich, Germany) and the recorded data were analyzed using a Brain Products Vision Analyzer. Latencies and amplitudes of the main components (N1, P1, N2) were analyzed in the form of absolute values and in the form of binary variables representing prolonged latency of P1 component, absent response of tSSEP pathology (absent response), and any tSSEP pathology (prolonged latency or absent response). Latency was considered prolonged if there was an increase of >2.5 standard deviations to the mean value for laboratory’s normative data.

In order to incorporate all tSSEP abnormalities in one variable we developed tSSEP score based on the information about prolonged latencies and absent response. Because we found no statistically significant differences in the amplitudes of the main components between healthy controls and MS patients in our previous work and because N1 and N2 waves due to large variability are often undetectable and cause irregular morphology, we excluded amplitudes from the tSSEP score analysis (12,13). The tSSEP score was calculated from the raw tSSEP data according to cut-off values for absent response and prolonged latency of the main component, P1 (0 = normal response, 1 = prolonged latency, 3 = absent response). It was calculated separately for the left (left tSSEP) and the right side (right tSSEP) and the two values were summed.

### MRI

All MRIs were performed on a 1.5T MRI scanner. Only brainstem multi-planar dual fast spin-echo PD and T2-WI sequences were analyzed for the presence of demyelinating lesions in the brainstem as a whole, and the midbrain, pons, and medulla oblongata separately. Furthermore, this involvement was then subdivided into right or left. If the lesion was located in the center of the brainstem, this was considered as bilateral, left, and right involvement. All MRIs were reviewed by two independent investigators (MH and LC), who were blinded to the patient’s symptoms at the time of analysis, and only lesions identified by both investigators were considered as present.

### Outcomes

The primary outcome of this study was to determine the prevalence of tSSEP abnormalities in patients with CIS, and to correlate these abnormalities with clinical symptoms and brainstem MRI.

Secondary outcomes were to determine whether the interpretation of the tSSEP results in the form of the tSSEP score enables better evaluation of the afferent trigeminal pathway involvement than analyzing each tSSEP parameter separately.

### Statistical analysis

Statistical analysis was performed using the IBM SPSS software, version 20 (Armonk, NY, USA). Values are presented as a mean with standard deviation or median with ranges. The Kolmogorov-Smirnov test was applied to test the normality of distribution. Differences in the distribution of qualitative variables were determined using the χ^2^ test, while the differences in quantitative variables were determined using the *t* test and Mann-Whitney test. Logistic regression was used in order to determine which variables were significant predictors for a specific model. *P* values lower than 0.05 or corrected with Bonferroni correction were considered as significant.

## Results

### Patients

We enrolled 115 CIS patients, mean age 32.5 ± 8.6, 82 (71.3%) women, with a median Expanded Disability Status Scale (EDSS) of 1.0 (0-3.5); 36 (31.3%) patients presented with optic neuritis, 36 (31.3%) with incomplete transverse myelitis, 27 (23.5%) with brainstem/cerebellar symptoms, 12 (10.4%) with hemispheral involvement, and 4 (3.5%) with multifocal involvement. Patients’ symptoms and brainstem MRI lesion distribution are shown in Table 1.

**Table 1:** Patients’ symptoms and brainstem magnetic resonance imaging (MRI) lesion distribution

	Present, N (%)	Absent, N (%)
Clinical symptoms		
overall	13 (11.3)	102 (88.7)
left	6 (5.2)	109 (94.8)
right	8 (7.0)	107 (93.0)
Brainstem MRI		
overall	48 (41.7)	67 (58.3)
left	33 (28.7)	82 (71.3)
right	33 (28.7)	82 (71.3)
Midbrain MRI		
overall	15 (13.0)	100 (87.0)
left	11 (9.6)	104 (90.4)
right	10 (8.7)	105 (91.3)
Pontine MRI		
overall	35 (30.4)	80 (69.6)
left	25 (21.7)	90 (78.3)
right	24 (20.9)	91 (79.1)
Medulla oblongata MRI		
overall	17 (14.8)	98 (85.2)
left	11 (9.6)	104 (90.4)
right	16 (13.9)	99 (86.1)

### Primary outcomes

Descriptive tSSEP values and proportion of patients with tSSEP abnormalities (prolonged latencies and absent responses) are presented in Tables 2 and 3. There was no significant difference in the absolute values of the tSSEP variables (latencies and amplitudes) between patients with and without clinical symptoms. Also, there was no significant difference in tSSEP variables for the right side regarding the presence of the right side MRI lesions. Patients who had MRI lesions in the left medulla oblongata had significantly prolonged latency of P1 component (22.9 ± 2.1 vs 21.2 ± 1.6, *P* = 0.006), while other variables did not show significant difference.

**Table 2:** Descriptive values for tongue somatosensory evoked potentials (tSSEP) latencies (lat) and amplitudes (amp)*

Variable (unit) (N)	Mean	Minimum	Maximum	Standard deviation
Right tSSEP				
N1 lat (ms) (81)	15.15	12.80	19.20	1.38
N1 A (µV) (81)	-0.65	-3.80	1.10	0.88
P1 lat (ms) (102)	21.48	15.40	26.20	1.75
P1 A (µV) (102)	1.39	-2.20	4.10	0.95
N2 lat (ms) (81)	28.32	24.20	32.20	1.92
N2 A (µV) (81)	-0.31	-3.40	1.40	0.92
Left tSSEP				
N1 lat (ms) (80)	15.16	12.40	20.00	1.47
N1 A (µV) (80)	-0.36	-2.30	1.10	0.70
P1 lat (ms) (100)	21.31	17.40	26.60	1.67
P1 A (µV) (100)	1.44	-1.50	4.30	1.05
N2 lat (ms) (79)	27.90	23.40	34.00	2.04
N2 A (µV) (79)	-0.24	-1.90	2.30	0.76

*13 patients had absent response for the right side stimulation, and 15 patients had absent response for the left side. N1 and N2 waves have large variability, due to difficulties with their detection.

**Table 3:** The proportion of patients with tongue somatosensory evoked potentials (tSSEP) abnormalities

	Present N (%)	Absent N (%)
Prolonged tSSEP latency		
overall	14 (15.2)	78 (84.8)
left	11 (11.0)	89 (89.0)
right	7 (6.9)	95 (93.1)
Absent tSSEP response (conduction block)		
overall	23 (20.0)	92(80.0)
left	15 (13.0)	100 (87.0)
right	13 (11.3)	102 (88.7)
Any pathology of the tSSEP response		
overall	37 (32.2)	78 (67.8)
left	26 (22.6)	89 (77.4)
right	20 (17.4)	95 (82.6)

There was no significant association between brainstem MRI lesions and clinical symptoms (Table 4). Furthermore, brainstem lesions (midbrain and pontine lesions specifically) were associated with the absent tSSEP responses (*P* = 0.002 and *P* = 0.005, respectively) (Table 4). There was no association between clinical symptoms and tSSEP abnormalities (*P* = 0.544) (prolonged latencies and absent responses; *P* = 1.00, and *P* = 1.00, respectively). In order to see whether these differences were left/right specific, we completed the analysis for each side separately (Table 5).

**Table 4:** Correlations between magnetic resonance imaging (MRI) parameters, clinical symptoms, and tongue somatosensory evoked potentials (tSSEP) pathologies (Bonferroni corrected *P*-value = 0.0125).

MRI lesions		N*	P*	*P* value
**Clinical symptoms**				
brainstem	N	63	4	0.040
	P	39	9	
midbrain	N	89	11	0.678
	P	13	2	
pons	N	74	6	0.062
	P	28	7	
medulla oblongata	N	88	10	0.405
	P	14	3	
**Prolonged tSSEP latency**				
brainstem	N	51	10	0.766
	P	27	4	
midbrain	N	72	13	1.000
	P	6	1	
pons	N	60	10	0.736
	P	18	4	
medulla oblongata	N	69	12	1.000
	P	9	2	
**Absent tSSEP response (conduction block)**				
brainstem	N	61	6	**0.001**
	P	31	17	
midbrain	N	85	15	**0.002**
	P	7	8	
pons	N	70	10	**0.005**
	P	22	13	
medulla oblongata	N	81	17	0.105
	P	11	6	
**Any pathology of the tSSEP response**				
brainstem	N	51	16	0.028
	P	27	21	
midbrain	N	72	28	0.019
	P	6	9	
pons	N	60	20	0.017
	P	18	17	
medulla oblongata	N	69	29	0.169
	P	9	8	

*N – negative, P – positive.

**Table 5:** Correlations between magnetic resonance imaging (MRI) parameters, clinical symptoms, and tongue somatosensory evoked potentials (tSSEP) pathologies regarding left and right side distribution (Bonferroni corrected *P* value = 0.0125).

Right					Left				
		N*	P*	*P* value			N	P	*P* value
**Clinical symptoms**									
brainstem	N	79	3	0.042	brainstem	N	79	3	0.352
	P	28	5			P	30	3	
midbrain	N	98	7	0.529	midbrain	N	98	6	1.000
	P	9	1			P	11	0	
pons	N	87	4	0.058	pons	N	87	3	0.116
	P	20	4			P	22	3	
medulla oblongata	N	93	6	0.308	medulla oblongata	N	98	6	1.000
	P	14	2			P	11	0	
**Prolonged tSSEP latency**									
brainstem	N	69	5	1.000	brainstem	N	69	6	0.136
	P	26	2			P	20	5	
midbrain	N	89	6	0.402	midbrain	N	85	9	0.129
	P	6	1			P	4	2	
pons	N	75	5	0.642	pons	N	76	6	0.025
	P	20	2			P	13	5	
medulla oblongata	N	83	6	1.000	medulla oblongata	N	84	8	0.041
	P	12	1			P	5	3	
**Absent tSSEP response (conduction block)**									
brainstem	N	74	8	0.516	brainstem	N	75	7	0.033
	P	28	5			P	25	8	
midbrain	N	95	10	0.085	midbrain	N	94	10	**0.006**
	P	7	3			P	6	5	
pons	N	80	11	1.000	pons	N	82	8	0.020
	P	22	2			P	18	7	
medulla oblongata	N	89	10	0.388	medulla oblongata	N	92	12	0.155
	P	13	3			P	8	3	
**Any pathology of the tSSEP response**									
brainstem	N	69	13	0.588	brainstem	N	69	13	**0.008**
	P	26	7			P	20	13	
midbrain	N	89	16	0.070	midbrain	N	85	9	**0.003**
	P	6	4			P	4	2	
pons	N	75	16	1.000	pons	N	76	14	**0.001**
	P	20	4			P	13	12	
medulla oblongata	N	83	16	0.475	medulla oblongata	N	84	20	0.016
	P	12	4			P	5	6	

*N – negative, P – positive.

### Secondary outcomes

tSSEP score was calculated for each patient, the median tSSEP score for the left side was 0 (range, 0-3), for the right side was 0 (range, 0-3), and overall tSSEP score was 0 (range, 0-6). There was no difference in tSSEP score between patients with and without clinical symptoms, however tSSEP score was significantly higher in patients with brainstem lesions, especially midbrain (*P* = 0.004) and pontine lesions (*P* = 0.008) (Table 6). In order to see whether these differences were left/right specific, we completed the analysis for each side separately, and found higher tSSEP score for the left side in patients with midbrain and pontine lesions (Table 7).

**Table 6:** Comparison of tongue somatosensory evoked potentials (tSSEP) score for different magnetic resonance imaging (MRI) lesions and clinical symptoms (Bonferroni adjusted *P* = 0.01).

		Mean rank	Median	*P* value
Clinical symptoms	0	58.57	0	0.536
	1	53.54	0	
Brainstem	0	52.40	0	**0.010**
	1	65.82	0	
Midbrain	0	55.17	0	**0.004**
	1	76.87	3	
Pons	0	53.49	0	**0.008**
	1	68.31	0	
Medulla oblongata	0	56.39	0	0.134
	1	67.26	0	

**Table 7:** Comparison of tongue somatosensory evoked potentials (tSSEP) score for different magnetic resonance imaging (MRI) lesions and clinical symptoms, separate for right and left side (Bonferroni adjusted *P* value = 0.01)

Right					Left				
		mean rank	median	*P*			mean rank	median	*P*
Clinical symptoms	0	58.27	0.00	0.329	Clinical symptoms	0	57.80	0.00	0.705
	1 54.38	0.00			1	61.67	0.00		
Brainstem	0	57.06	0.00	0.470	Brainstem	0	54.04	0.00	**0.006**
	1	60.33	0.00			1	67.85	0.00	
Midbrain	0	56.72	0.00	0.044	Midbrain	0	55.38	0.00	**0.000**
	1	71.40	0.00			1	82.73	1.00	
Pons	0	58.18	0.00	0.867	Pons	0	53.93	0.00	**0.001**
	1	57.33	0.00			1	72.64	0.00	
Medulla oblongata	0	57.25	0.00	0.364	Medulla oblongata	0	56.12	0.00	0.011
	1	62.63	0.00			1	75.82	1.00	

Finally, we performed binary logistic regression in order to investigate the effect of tSSEP score on the likelihood that patients have midbrain MRI lesions. The logistic model was significant, χ^2^ (1) = 6.804, *P* = 0.009. It explained 10.7% (Nagelkerke R2) of the variance in presence of midbrain MR lesions and correctly classified 87% of cases. The tSSEP score was a significant predictor for midbrain lesions (*P* = 0.007, Exp(B) = 1.466).

## Discussion

Two main findings of this study are: 1) tSSEP abnormalities are associated with midbrain lesions in CIS patients; and 2) the tSSEP score is a significant predictor for the presence of midbrain lesions in CIS patients.

In recent years there has been an increase in interest in neurophysiological assessment of MS patients despite the dominant role of MRI in the disease diagnosis and monitoring. This is mainly due to two reasons. First, as already mentioned, standard MRI sequences (T2, FLAIR, and T1 after application of gadolinium) lack the sensitivity for detection of demyelinating lesions in the posterior fossa. Second, MRI is used solely for morphological assessment and does not provide information on functional integrity of the presented structures. Connor et al (14) demonstrated that multimodal evoked potentials were useful as an instrument of measuring clinical disability in MS. Further studies provided evidence that evoked potentials may also play a role in predicting disability in MS patients (9,10). However, these studies assessed brainstem dysfunction using only brainstem auditory evoked potentials (BAEP), a method that evaluates just one of the numerous pathways in that area of the central nervous system. Additional mode of brainstem assessment is the vestibular evoked myogenic potentials (VEMP), which have shown to be of value in the evaluation of brainstem involvement in MS (15).

Previous studies have shown that MS patients have significantly prolonged latencies on tSSEP compared to healthy controls (12,13,16). The present study showed that tSSEP abnormalities were associated with midbrain lesions. The trigeminal nerve has three principal sensory nuclei: the primary sensory nucleus (touch and position sensation), mesencephalic nucleus (proprioception), and the spinal nucleus and tract (pain and temperature sensation). With the intrapontine fascicular part of the trigeminal nerve they form the brainstem part of the trigeminal afferent pathway (5). MRI studies have shown that the intrapontine fascicular part of the trigeminal nerve is most frequently affected, while the lesions in the spinal nucleus and tract and the intrapontine fascicular part of the trigeminal nerve correspond with trigeminal neuralgia (5). On the other hand, in none of the published studies pain, temperature, and/or touch sensation correlated with the presence of brainstem lesions (17). Investigating proprioception of the face is very difficult (18), and no studies have been performed on MS patients. The consistent finding of the present study was the association between tSSEP and midbrain lesions on the MRI, indicating that tSSEP evaluates proprioception of the face. This could explain no association between patient-reported symptoms and tSSEP results. Initial studies of the trigeminal nerve SSEP have proven to be an objective, non-invasive measurement of facial proprioception (19,20), and our study confirms these observations with an excellent neurophysiological-MRI association. Further studies investigating the clinical involvement of facial proprioception and tSSEP are warranted. Because the aim of our study was to investigate the tSSEP changes in relation to brainstem MRI findings we wanted to investigate if there were any side specific associations between the tSSEP variable for the specific side and the MRI lesion on the same side. The reason why statistical significance was obtained only for the left side could be explained with higher numbers of tSSEP pathologies on the left side.

Another aspect of the study is the interpretation of the evoked potential results in the form of the score, because this method enables more comprehensive evaluation of the observed changes. We developed the tSSEP score calculation on the basis of prior evoked potentials studies (21). The tSSEP score was significantly higher in patients with brainstem lesions on the MRI, specifically midbrain lesions for each side. Studies that have used BAEP as the method of choice for evaluating brainstem dysfunction did not find it to correlate well with clinical symptoms (9). Some studies have indicated that VEMPs could replace the BAEP, as it shows good correlations with the EDSS (14). Thus, the important finding of this study is that tSSEP represents a brainstem evoked potential test that shows good association with the presence of demyelinating lesions on MRI, supporting its use in MS evaluation. Future studies combining different brainstem evoked potentials in the form of scores may prove to be useful in monitoring and/or predicting disease activity in MS patients.

This study has several limitations. The first is that all MRI were performed on 1.5T MRI. However, although 3T MRI provides higher sensitivity in the detection of MS lesions, especially in the infratentorial region, it is not widely available. Furthermore, although healthy controls were not included in this study, we used the reference values of our laboratory.

In conclusion, this study establishes the value of tSSEP in evaluating brainstem function in CIS. As there have been several studies evaluating evoked potentials as a prognostic method, future studies should include tSSEP as a valuable addition.
